# *My Pillow Is Filled with Tears…* Syrian Refugees’ Journey to Australia: Narratives of Human Courage and Resilience

**DOI:** 10.3390/ijerph22050691

**Published:** 2025-04-27

**Authors:** Rosemary Qummouh, Sheridan Linnell, Shameran Slewa-Younan, Sera Harris

**Affiliations:** 1 School of Social Sciences, Parramatta South Campus, Western Sydney University, Penrith 2751, Australia; s.linnell@westernsydney.edu.au (S.L.); s.harris@westernsydney.edu.au (S.H.); 2 School of Medicine, Macarthur Clinical School, Campbelltown Hospital, Western Sydney University, Penrith 2751, Australia; s.younan@westernsydney.edu.au

**Keywords:** displacement trauma, refugee mental health, transit, resettlement trajectory, resilience, intersectionality, Syria

## Abstract

This article showcases Syrian refugees’ narratives of trauma and survival, through a phenomenological approach to in-depth research, with refugees who have resettled in Australia. It explores their journey towards resettlement, highlighting the nexus between displacement in the home–transit–host countries and the biopsychosocial determinants of mental health. Since the 2011 uprising, over 12 million Syrians have been displaced, both internally and worldwide. A refugee’s journey to safety often involves multiple displacements and exposure to dangerous, life-threatening, and dehumanising experiences. We have therefore adopted a qualitative approach that counters this dehumanisation by honouring the unique humanity in the voice of each of our research participants. This article aims to portray the nuanced interdependence between the individual, social, and political contexts of seven Syrian refugees’ lived experiences through an in-depth consideration of what they have told us, how they narrate their stories, and the meanings they ascribe to what they have experienced. The findings of this small yet eloquent study reinforce the insight that the journey to resettlement is far from linear and that resettlement itself is a process marked by recurrent and persistent complexities. The article suggests that the resilience of these refugees is best understood as an ethical and altruistic commitment to collective well-being, transcending notions of individual fortitude.

## 1. Introduction

Syrian people’s displacement began in 2011 due to the Syrian Civil War’s uprising phase. Syria’s involvement in the Arab Spring, following Egypt and Tunisia’s demonstrations to overthrow their governments and end 40 years of oppression, ended with millions of Syrians displaced both globally and internally. Syrian refugees continue “to represent the refugee population with the highest global resettlement needs” [1], with the United Nations estimating that 6.8 million Syrians have been forcibly removed from their homes, communities, and land and are displaced within Syria, being the highest internally displaced number in the world and the highest since the conflict started in 2011 [1,2]. More than 6.6 million Syrians have been forced to flee their country due to political unrest and seek protection and safety in neighbouring countries such as Lebanon, Jordan and Turkey. Simply put, this is far from over; the Syrian crisis is presently in its 13th year, and the UNHCR has estimated that “over 753,000 Syrian refugees are projected to be in need of resettlement in 2024” [1].

Millions of Syrians seeking asylum or applying for refugee status because of persecution based on religion, race, or political opinion have caused an unprecedented increase in the number of displaced persons seeking protection [1]. Many Syrians in their transit initially claimed temporary protection in neighbouring countries before applying for refugee status under the protection of the UNHCR or in the context of this study through the Australian Government’s Refugee and Humanitarian Program. They were, and millions are still, held in transit countries, such as Lebanon, Jordan, Turkey and Greece, in limbo, waiting for refugee status in either the transit country or other host countries such as Australia [3]. They face complex and challenging situations threatening their fundamental human rights of survival and safety, education, employment, primary healthcare, and housing [4]. Kira et al. (2017) discuss the complexities and significant impacts of these threats on refugees’ fundamental human rights, leading to an increased prevalence of mental health disorders such as depression, PTSD and anxiety [5]. The prevalence of these serious mental health problems among Syrian refugees in transit countries with already over-burdened health systems presents complex public health challenges that require a coordinated and nuanced response [6].

The search for safety and security comes at a great cost for the Syrian refugees transiting in Lebanon, Jordan, and Turkey. UNHCR Lebanon (2024) estimated that 90%, which is about 1.5 million of the Syrian refugees displaced in Lebanon, are living in extreme poverty and vulnerable hostile conditions. Moreover, the collapse of the economy in Lebanon has severely impacted both the Syrian refugees and the Lebanese people, leading to the heartbreaking exploitation of the marginalised [7]. In Jordan, Syrian refugees are displaced and dispersed throughout the country, with over 670,000 living in private rentals exposed to exploitative inflated rental costs [1]. In Turkey, unprecedented numbers of Syrian refugees are faced with involuntary returns to Syria due to a recent change in Turkish government policy. In these circumstances, World Vision (2024), UNHCR (2024), and Human Rights Watch (HRW) (2021) all report an imperative need for continual humanitarian aid and provision to Syrian refugees [1,2,8]. This situation highlights a crucial need for understanding the widespread effects of the crisis on individuals and communities and the psychological and emotional consequences of displacement.

Syrians who sought refuge in these neighbouring countries are being slowly returned to a fractured and unrecognisable Syria devastated by the Civil War. Lebanon has returned 10,130 Syrian refugees in 2023 [1]. Since December 2023, HRW (2024) reported that 57,519 Syrian refugees have been deported from Turkey back to Syria, and Jordan has returned 2600 Syrian refugees in 2023 [9]. The Syria UN Commission of Inquiry established that Syrian refugees are at significant risk of “arbitrary arrests, detentions, torture, mistreatment, involuntary or enforced disappearances and summary executions” [8]. This is a vital concern, especially considering that countries like Lebanon and Jordan have not ratified the 1951 Refugee Convention. Nevertheless, Lebanon is currently hosting the highest number of Syrian refugees per capita, while Jordan is home to the second largest Syrian refugee camp, following Turkey [7,10].

Since 7 October 2023, the Israel–Palestine conflict has brought to the forefront a further humanitarian catastrophe, impacting neighbouring countries such as Lebanon and Jordan, where hundreds of thousands of Palestinian refugees have sought refuge in an already overpopulated and dire situation [11]. Israel attacking the South of Lebanon has resulted in thousands of Lebanese civilians and Syrian refugees in the area fleeing from their homes into other parts of Lebanon [9]. This brings further pressure on first asylum countries (transit), which “have shown incredible hospitality for over a decade [and] are now suffering from layers of their own in-country crises, which has led to an increasingly challenging protection environment” [1]. Meanwhile, André (2023) questions the retreat from a rights-based paradigm toward a narrow, individualistic concept of resilience, underscoring the “clear detrimental consequences of neoliberal governmentality for the living conditions of Syrian refugees” [12] (p. 57).

### The Focus of This Research

While emergent literature speaks to the importance of refugee settlement experiences in host countries, specific psychosocial and cultural determinants of post-traumatic stress or resilience appear not to have been described in the literature on Syrian refugee experiences, with insufficient emphasis on capturing earlier traumas from home and the trajectory of traumatic experiences from transit to host [13,14,15,16]. Furthermore, no specific studies exploring the impacts resettlement has on Syrian refugees’ mental health, psychosocial needs, or resilience through the three-stage trajectory have been found in the literature from an Australian context [17].

The introduction to this article frames the scale and complexity of the issues facing Syrian refugees. As this article will argue, their journey to resettlement is neither simple nor linear. The journey can be long and complex, often described by the refugees interviewed as involving experiences of multiple dangerous stops along the way. Crucially, this article showcases the refugee participants’ narratives of trauma and survival, highlighting the nexus between displacement and the biopsychosocial determinants of mental health through a phenomenological lens and unpacking specific experiences of one of the largest humanitarian crises the world has witnessed.

## 2. Materials and Methods

A pragmatic approach using Interpretative Phenomenological Analysis (IPA) was applied. Phenomenology is a qualitative method focused on exploring individual lived experiences and meaning-making. To ensure that phenomenology is reliable in investigating the lived experiences of research participants, Polkinghorne (1989) advises that “phenomenological researchers should interview between five and ten participants who have all experienced similar events (phenomena). As such, the commonality of their experiences can be captured and interpreted” [18] (p. 2).

The Syrian refugee participants were recruited from the Greater Western Sydney local government areas of Fairfield and Liverpool in NSW, Australia. These areas have many Syrian refugees and established settlement services catering to their community language (Arabic) [19,20].

### 2.1. Method

Congruent with the emphasis on the importance of lived experiences, IPA recommends the use of semi-structured interviews [18]. This approach enabled the research to explore the impacts of (re) settlement, understand the human resilience factors associated with trauma environments and examine the impacts contributing to resilience and mental health well-being from the perspectives of those who experience these phenomena as they reflect on them post-trauma [21].

A key advantage of the IPA approach is “allowing the interviewees to express themselves and their lived experience stories… without any distortion and/or prosecution” [18] (p. 9), which is crucial to the ethics of conducting research with refugees [22]. A strong relational emphasis was particularly important, given that the refugees who participated in this research have been subjected to multiple displacements, and being ‘questioned’ may have negative associations.

### 2.2. Participants

Seven Syrian refugees were interviewed. To meet the study’s criteria, participants needed to have claimed asylum in neighbouring countries before applying for refugee status under the protection of the UNHCR or through the Australian Government’s Refugee and Humanitarian Program applications made to the Australian Department of Home Affairs (refugee visa 842 with family in Australia as proposer if applicable) [23,24].

### 2.3. Data Collection

The semi-structured face-to-face interviews ranged between 60–75 min. Participants were given an open-ended invitation through the initial question, “What is your story?” before being prompted to expand on their experiences in Syria, in transit, and in Australia, focusing on the context, the challenges, and what helped them cope. In the tradition of IPA, the interview questions were framed to elicit individual responses that would contribute to a rich picture of the diversity of Syrian refugees’ experiences as well as to the emergence of commonalities [25].

### 2.4. Ethical Considerations

The study was approved by Western Sydney University’s Human Research Ethics Committee (H15267). All participants signed informed consent forms before the interviews. Pseudonyms were used to maintain participants’ anonymity. There were culturally appropriate supports and resources available for any participants who might need them. Interviews took place in participants’ preferred languages and locations. Ethical considerations extended beyond these formal requirements of ethics approval. There was an overarching ethical intention to conduct this research in ways that acknowledge and clearly oppose the humiliations and structural exploitation to which refugees are frequently subjected. The project adopted an iterative approach to informed consent, and the first author engaged in ongoing consultation with the participants about their experiences of the research process and outcomes. The authors were mindful of honouring the expertise and agency of participants and the importance of checking in with them, as well as reflecting with each other throughout the conduct of the research.

### 2.5. Data Analysis

Thematic analysis was used to code interview transcripts, and the content was analysed using an interpretive framework to convey the depth of participants’ experiences and identify the major themes present in the data. This approach allowed for recurring patterns of meaning to emerge to reveal commonalities across participants’ responses. Emerging themes were identified from the multiple ‘deep dives’ into the complete transcriptions of the refugee participants.

## 3. Results

Three central themes were identified: firstly, multiple challenges marked their displacement journey, including existential anxiety, psychosocial stressors, and economic and demoralising events. The participants were impacted by experiences of discrimination, exploitation and targeted attacks originating in Syria and reinforced while in transit. These experiences had an influence on the complexities of participants’ resettlement in Australia and on their mental health. Secondly, the participants’ survival depended on cultural context, privilege, and determination as a result of their stress and stress responses to the challenges and trauma experienced. Thirdly, narratives revealed how a collectivist rather than individualist sense of identity persisted across the displacement stages, demonstrating a significant shift from Western understandings of refugee trauma and mental health. More specifically, there is no adequate one-size-fits-all approach to resettlement [26]. This key finding of a collectivist sense of self and identity, seldom thinking about themselves in isolation but identifying as belonging to a broader family and community system, underpins the research findings and extends them beyond conventional notions of trauma or resilience. The themes are interchangeably reflected below.

### 3.1. Challenges and Experiences of Multiple Displacements

To understand the challenges and complexities of the participants’ lived experiences, it was crucial to unpack their narratives, focusing on coping skills throughout their journey. This led to conversations surrounding their emotional and psychological passage as refugees, shedding light on the difficulties and struggles they faced during the different stages of their displacement. These stories highlighted shared experiences, which included traumatic events during initial displacement as internally displaced in Syria, personal accounts of survival and coping in both Syria and transit, and the biopsychosocial impacts that their displacement journey had on them once safety was reached.

It is important to begin this section of the paper with an account of the deep and abiding connections Syrian refugees express with their homeland. Participants shared their memories, nostalgia, and a deep sense of loss for the Syria they once knew. There is not a single refugee the first author has worked with who does not narrate their homeland’s beauty with warm smiles, heartfelt sentiments, and hopes for a return to how their lives once were. As participant Zohra reflects:

*“[L]ife was stable, and we were living with family, a wonderful life, and I was thinking of the future that I even bought a piece of land so I can create a house in a small farm in our village.” and for participant Alfred:*

*“We still hold on to the memories and nostalgia for our country.”*

### 3.2. The Conflict

The results, as shown in Table 1, indicate that the participants spent an average of 6 years in the conflict, and all described this time as playing a game of ‘Russian roulette’ that Syrians braved and risked daily to survive. They all shared how they were open targets, constantly vulnerable and susceptible to kidnappings, receiving threats of rape, and the unknown dangers that awaited them.

Particularly revealing is how Zohra described it this way:

*“The mortar shells were constantly being fired at us, especially targeted towards schools, markets, and areas where large numbers of people gathered were hit the most. At that time…we were telling ourselves that it was impossible for the war to continue and that it would end, and the army would win until events developed dramatically to the point where, in fact, we felt that there was no safety, and the situation of the war was coming closer to us, and we were in very great danger.”*

Death and dying became a standard narrative in the lives of the interviewed Syrian refugees, and they questioned their existence and hope of survival. All seven participants recollected the numerous times they were forcibly internally displaced until they escaped to transit as being short-term solutions in the hope that the war and conflict would soon end. During these trying times, the participants questioned their existence, bargaining with anything from which they could draw strength, such as praying and family. As participant Zohra reflected:

*“Every morning, I send the kids to school (in Syria), and my heart wonders if this may be the last day that I will see them because a lot of my students and friends have died and lost their children have died in the market, on the street, at school, nowhere was safe.”*

Further adding…

*“We would hear every day that 50 people had died, 12 people had died. And I would say to myself, is it reasonable that we, one of these days, as a family, become one of these numbers mentioned in the news that 20 people were killed as a result of a mortar shelling? Is it possible that we are just a number, and my name is not mentioned or remembered? Life was very difficult.”*

Mona reflected:

*“We used to sleep on explosions and wake up with the explosions, the shooting; you would see that we were not comfortable at all, not psychologically nor…there was a level of fear that was not normal… They used to say we will kill you, and we will not leave you, so threats to scare us. And it did really happen. They went into the village, and I told you about how they kidnapped some of them, and after they kidnapped, we thought, that’s it.”*

For Yusra, she reflected:

*“a number of times we were subjected to incidents, like missiles hit over the top of us and our schools, and my father was kidnapped for a period… It was scary. We were even scared to walk in the street because we are scared someone will kidnap us or do something.”*

For Alfred:

*“No one was well anymore, old and young, rich and poor. We went to Lebanon to escape death.”*

Maryam and her family experienced near-death experiences on several occasions in Syria. Maryam’s husband was kidnapped for three days, and she described these three days as *“a very difficult time, and we experienced events that would need twenty years to heal ourselves. How we put up with it and kept going, I don’t know”*. This at the back of threats of her two daughters being kidnapped and raped, being victims of street bombings meters from where they were standing, and constant threats of militia invading their home; describing the only way she survived these threats is to arm herself, viewing death and dying to be as dignified as possible:

*“I will never forget how we lived through the five hours of the threat that the terrorists will enter our area. Last time, it was very difficult. Five hours passed, and I don’t count them as part of my life. This time, I decided that if it happened again and they attacked our home, I would blow myself up and my daughters before anyone assaulted us. Insecurity, the difficult economic situation, the feeling that you are in your own country threatened and with no security, and being vulnerable Christians, everyone telling us that we support the government made us feel that we do not live in our country and that we are hated by everyone.”*

For Yusra, the frustrations experienced by being vulnerable and overcome with debilitating fear took away opportunities for any normalcy:

*“We were going to another area; we were waiting for the bus to go. A missile hit the building that we were standing underneath… I will never forget that because we started running, it was very difficult, yes. It was not just about being scared; you are also upset because you can’t live your life like any normal person is living.”*

Yet, even in participants’ stories of being in the midst of conflict, care for others began to emerge as a sustaining force. Maya described how her fears for her children eclipsed any fear for herself:

*“We thought that is it, the armed groups have come to us. When they say armed groups are going to come into your house, what do you imagine, your daughter? What do you imagine your son? What do you imagine? I swear to God that you are not scared for yourself.”*

### 3.3. Transit

The threats did not end with transit into neighbouring countries. Participants shared stories filled with how every aspect of their existence was controlled, degraded, humiliated, and filled with social injustices and discrimination both at home and in transit. They lived in absolute fear that, at any moment, while they were in transit, they could get arrested or deported back to Syria just for being a Syrian refugee.

Participant Alina tells of the degrading and often demoralising experiences that were characterised by existential anxiety while in transit:

*“We were not stable, not emotionally or psychologically, and not in our living there (Lebanon), we were always threatened as Syrians, that Syrians go back to your country and our country had no stability. There was a war, there were terrorist groups that we were threatened by, and so we could not return, so we were always living on the edge that the Lebanese would kick us out, and we would have to go back to our country.”*

Four of the seven participants who transited to Lebanon reflected their time as filled with daily challenges to maintain survival, particularly in a country such as Lebanon, where there is a history of conflict between the two nations spanning almost three decades (Lebanese Civil War 1975–1990) and reported the impacts on their mental health and their children’s well-being. They experienced multiple threats of deportation, lack of access to education and employment opportunities, sexual harassment, and beatings that carried over into their settlement in Australia.

Alina described it this way:

*“The situation between the Lebanese and Syrians was not good. Because there were always lots of laws that were being passed that, the Syrian couldn’t leave the house after 5:00 pm. And so, you would see there were people hitting the Syrians just because the Syrians were in the street.”*

Alina spent the longest time in transit compared to other participants, holding onto fear for seven years while in Lebanon:

*“So that is why in Lebanon we were always in a state of fear because of the laws that were being passed by the Lebanese that the Syrians need to go back to their own country and the Syrians need to be deported. Things like that, we were always in a state of fear; there was no stability.”*

Alfred nevertheless holds out hope that the current pressure to expel Syrian refugees from Lebanon might shift toward an understanding of the commonality of both peoples’ suffering:

*“God willing, these countries will sympathise with us and see the suffering of the two peoples. Even the Syrian refugees in Lebanon, now they are trying to return them, and some of them do not want to return.”*

Particularly revealing of this bare reality is how Alina described the hardship experienced during transit:

*“We slept on a piece of metal, the metal of our bed. There was no mattress on it, there were no things, there was nothing. We had been promised work, but the person who promised us work was a liar, and the money that we had ran out, and we didn’t have any food left or money or any work. So, we went through a degrading time.”*

However, when Alina did find work, the degradation continued through the sexual harassment she had to endure in successive workplaces:

*“I changed a number of jobs. One of them was in the sweet shop and cleaning, and one was doing the accounts for a pizza (manaish) shop because most of the work that I was going into there would be sexual harassment. So, from the owner of the house or the owner of the work. So, he would either want me to be his girlfriend so that he can help me in work and give me a good amount. I had to fight with him and then stop work, so approximately three times I worked and three times the same thing happened to me. So, I would then leave work, and I wouldn’t even take what was rightfully mine; I would still have money with that person, but I wouldn’t take it, and I would say, just leave the money for you; I don’t want it. And I would leave work because of that.”*

These experiences were not uncommon for refugee women in particular. Maya expressed similar experiences of being sexually harassed and not being paid in Erbil:

*“I worked with my daughter… we went and worked for someone in a laundry, washing clothes and that and folding clothes for hotels and things like that. We worked for two weeks, and it was very bad, and he didn’t give us any money… he wanted me to take a day off, and she (daughter) take a different day off. He would not let us take the same day off, so I said to him, I am here for them; I have come here for their sake. So, if anything happens to them, I will be regretful all my life, no… there was that fear… they think that with money they can buy whatever they want, but of course not.”*

Maryam narrates the toll multiple displacements had on her two daughters:

*“The girls became depressed. They had no friends and did not know why they were there, what are they waiting for and whether Australia would grant us a visa or if we stayed in Erbil. And if we leave, it is another life. ‘How many times will we change our lives and change our homes?’, they would say.”*

She points out that transit is not an experience of freedom:

*“We emerged from the suffering of the war in Syria and arrived in Erbil and lived the suffering of different people, language, restrictions, and lack of freedom. You are not free.”*

All participants described experiences and impacts from home to transit countries. What stood out were the mental health impacts of demoralisation amplified by discrimination, exploitation, and racism endured in transit, further exacerbating the trauma they had experienced in Syria.

As participant Maya describes:

*“It is as if we have now become broken. It does not matter anymore from this house to that house because the first time you feel that it is very difficult, but then afterwards you feel that it is just normal. It is something that you have lost, that you have a house, you have privacy, something that’s yours, and you have that. Everything that you built up in 20 years has gone.”*

### 3.4. Resettlement

The narratives were all filled with complex day-to-day struggles, challenges, and discrimination while also dealing with more profound existential stressors, guilt, and grief, contributing to biopsychosocial impacts, such as chronic complex pain syndromes, high blood pressure, post-traumatic stress, depression, and anxiety. Participant Alina described the complexities as:

*“I was psychologically devastated, broken… the war and this psychological shock… I haven’t overcome anything, it’s still all there. When I came to Australia, everything was still there… the sense of security that I was living in, so straight away, we became the opposite. There was no security, there was no stability, there was no social life. There were no friends; there was nothing like before.”*

Maya also describes how the trauma she went through continues to haunt her:

*“The things that we went through are very difficult, and I came here (Australia), and I also need to remove all of these thoughts from my head, but I can’t remove them. I say to myself that I need to relax my thinking because we are very, very fatigued.”*

Other participants, for example, Zohra, similarly described the ongoing toll on their own and their families’ mental and physical health:

*“It is the anxiety… My husband has high blood pressure and started taking medication, and our psychological and mental health suffered, and we started having eczema. The stress, lack of sleep, and tension, and worry began to affect us psychologically and physically, and my hair began to fall out. I also lost a lot of weight, up to 10 or 15 kg.”*

Alfred described the trauma of the conflict in Syria as the main contributing factor to his wife’s cancer diagnosis several years later:

*“My wife was a schoolteacher and was attacked. They attacked the school, and she faced an almost certain death, which affected her psychologically, and she became suffering from constant stress and constant fear. Extreme stress and fear in her everyday life until now. She had a lump here (pointing to the throat area) … it turned out that this lump was cancer, but in the early stages… Thank God, the surgery she had four and a half years ago was successful.”*

Other devastating and incomprehensible losses underscore this gratitude for his wife’s recovery:

*“My mother passed away, and my sister, who was a year younger than me, also died, and she had no health issues… two years after her death, her twenty-year-old daughter also died. I went out into the street like a madman and said what a cruel life this is. A 20-year-old girl dies in mysterious circumstances from oppression, overthinking, and torment.”*

Despite the bleak narratives of what participant Maya expressed as *“fear that can’t be described”*, the intersectionality of honour, survival, and dignity shaped each personal account, exceeding conventional notions that resilience is an individual capacity for ‘bouncing back’. Each participant’s positionality foregrounded how they were sustained by their commitment to family members, highlighting altruism as a central, culturally nuanced construct:

*“My daughters were the main motivation, and if it were not for them, I would have died and committed suicide.”* *Maryam*

### 3.5. Connections

When the participants were asked about their emotional and psychological experiences during their journey as refugees, the stories they shared had distinct characteristics in common. Including, participants’ deep connection with and loss of their homeland appears to have elicited a deep connection with human suffering and loss, delicately linked to an altruistic sense of cultural identity and belonging. A life that was once filled with joy, safety, and security is now filled with so much loss, pain, sorrow, and mourning. Going through the analysis, the authors distinguished that the two aspects of loss and connection could not be isolated from each other or from the participants’ extraordinary capacity to keep going. Their love for Syria and longing for a sense of security, family, and community is both a measure of loss and the basis for their enduring social and spiritual resilience. Immense suffering is linked to an extension of an altruistic self [27]. It is better explained as post-traumatic growth, a term frequently used in the literature to explain this phenomenon. Acar et al. (2021) state, “a traumatic event that severely challenges and shatters the individual’s assumptions and beliefs about the world does not directly cause growth, but the emotional struggle following trauma is crucial for post-traumatic growth to be experienced” [28] (p. 2).

The findings also indicated a positive relationship with spirituality as an important source of sustenance in these narratives. This was expressed in different ways. For example, when Yusra reflects on the day-to-day experience of just trying to survive, spiritual gratitude for each new day is embedded in a phrase that in Western secular contexts is sometimes used lightly. Through the reverent tone and cultural context of these words, the first author could identify a sustaining spirituality that shines through. *“We just tried to live; it wasn’t more than just trying to live. So just get through the day and we would say thank God that we woke up to another day”*.

Maryam reminds us of the enormity of what she, her family, and her community faced and how her belief in God sustained her by saying, *“I always remember and wonder how we were able to handle all this and continue. How God stood by us until we passed all the trauma and war. Our life was perfect, and suddenly, we lost everything.”*

### 3.6. A Complex and Non-Linear Journey

The study found no linear way to understand or process the journey refugees take toward reaching safety and resettlement. This nexus came through the participants’ narratives once they reached safety in Australia. For example, Alfred eloquently describes this nexus through the metaphor of the roots of an oak tree, describing resettlement in Australia as:

*“It is hard when you are 57, and you move like an oak tree from its land and put it in another land. It takes time to adjust.”*

The wisdom of age-old cultural metaphors was significant in shaping participants’ narratives. In Arab worldviews, the pillow is a deep-seated representation used in language to connect a person to home, family, culture, identity, and belonging. For example, as Maya reflects:

*“How hard is it for you to leave your house, the house that you built, and you put all your effort into it, and you finished it, you worked hard, and just leave it for nothing. And then you sleep on a pillow that you do not know.”*

Alfred takes up the metaphor of the pillow as he narrates his deep sorrow about leaving family behind and the guilt over the real possibility of not seeing family again:

*“My tears are on my pillow every day; they would see me in another world, and I remain sad. Even though I would laugh and smile as a way to do something about my sadness, the truth is hard.”*

The narratives paint a lonely and painstaking picture of intense grief and loss for a deteriorating humanity and for those family members and communities left behind. A sense of ‘survivor guilt’ and the terror of threats to loved ones tinged many of the accounts, as Zohra conveyed:

*“They talked a lot in front of me and threatened to kill me, but they said they had pity on me because I was a Christian; [but] your children will be killed.”*

The participants’ narratives highlight a dire situation that they feel unable to escape from, encompassing the intersectionality of guilt, survival, and trauma. Physically, they are safe; however, mentally and psychologically, the war is not over. Knowing that family left behind in Syria, thirteen years on, still holds brutal consequences of the civil war and for safety. As participant Alfred reflected:

*“I am a man who loves my country, and this is how I raised my children. I was impacted a lot; in the end, there was nothing I could do about the situation; this is reality.”*

Participants in the study described the psychological toll of holding onto trauma from their home and, through their journey, carried over into all aspects of their personhood. Alina described this psychological toll as being ‘broken’. Similarly, Maryam’s account underscores the ethical necessity for this article to bear witness to the extent of the suffering:

*“We were destroyed. Your mind needs to comprehend this.”*

### 3.7. Collectivist Versus Individualist Identity Persisted Across the Displacement Stages for the Participants

This key finding extends understanding of both trauma and resilience, highlighting the interdependence of shared cultural identity and mental health of Syrian refugees. This identified theme throughout the findings focused on survival as belonging to a collective sense of cultural identity, an extension of one’s self as opposed to an individualistic sense of self and identity [5,29]. All seven participants described how they survived their marginalised experiences throughout the displacement stages as being based on an altruistic sense of protection for their families. Particularly revealing is how participant Mona described it:

*“All the terror I lived through, I lived through it with my sick sister, so it was difficult from both sides; for me, it was hard that my sister was sick and with me, and it was difficult because it was a war. So, I was very scared, and I was scared for my sister…… And then my siblings said to me you have to leave; you have to for the sake of your sister because she is sick, and so we left.”*

Since refugees often have a collectivist cultural worldview, it is unhelpful to view resettlement from an individualist perspective. The participants in this study directly and implicitly linked their survival to being surrounded by their strong ties with their families as being a part of a cultural collective community that supports coping and recovery.

However, a collectivist identity may also exacerbate ‘survivor guilt’. All the participants pondered the consequences of their forced decisions to leave their homes. Almost all the participants in this study narrated, “What have I done?” This manifested as a loss of identity, guilt, and self-blame, tying it to their family’s future and uprooting to another country.

This feeling of losing one’s identity has been described by Alfred as:

*“I struggled for six months between me and myself, asking myself ‘What have I done’ and because of the different culture and language here. Life here is completely different. Social life is non-existent in Australia. I had countless contacts from a distinguished society, all of whom were university professors, teachers, and principals. My life was full. I worked as an accountant in a hotel where delegations used to come. As well as my small community of family and friends. I’m a social guy, and I like to socialise, but I’m losing all of that here.”*

Mona described her feelings of guilt based on her sick sister’s last words:

*“She wasn’t happy, she would say I don’t want to leave this home, this home is my father’s, and I don’t want to leave it… So now I still remember these words, and I get upset.”*

As for Maya, her feelings of self-blame told how:

*“We hated ourselves because how difficult is it the first time you leave your house, how hard is that you feel, what have I done to myself? My daughter is in university; what have I done to myself? I would always keep thinking about this… It’s because you’re thinking about your child’s future, about your daughter’s future, you’re not comfortable.”*

### 3.8. Stories of Hope and Resilience

The participant’s narratives paint a confronting picture; however, the study also found that all the participants used their trauma to motivate survival and understand their symptoms of post-traumatic stress, depression, anxiety, and grief while still holding their lives together through their cultural collective identities, their families and their strength.

Zohra identified it this way:

*“This is what makes me strong and enduring and not lose my balance and sanity. Maybe if I gave up and couldn’t bear it, I might become depressed… I thank God my optimism in life, my mentality, and my outlook prevented me from suffering from depression, even though I went through very difficult circumstances and a lot of fear, anxiety, and sadness, but thank God, my prayers, faith, caution, love for my children, and love for life made me get here.”*

The narratives personified notions of courage and hope despite the complexities of circumstances. They are true testaments to, and undeniably powerful reminders of, the human spirit’s strength and ability to overcome even the most challenging of circumstances.

A quote often credited to Khalil Gibran could not sum this up more perfectly.

‘Out of suffering have emerged the strongest souls; the most massive characters are seared with scars.’ [30].

## 4. Discussion

While the study aimed to explore the direct relationship and influence between multiple displacements and the impacts on a refugee’s mental health and resettlement, the findings showed that the journey is not straightforward. Instead, it was identified as multidimensional, with no clear separation across the three stages of displacement. However, the findings together highlighted the role of these experiences from Syria and while in transit, which are directly associated with the psychosocial complexities surrounding resettlement in Australia.

The study found that the participants’ narratives all featured extraordinary resilience and endurance despite the adversity and marginalisation throughout the multiple displacement stages. Participants’ stories, however traumatic, continuously raised questions surrounding existential positionality. The focus of their lived experiences was not just on survival but also on navigating the complexities spanning an average of six years in conflict, a further average of four and a half years in transit, and the traumas the participants have endured until reaching safety, as well as the ongoing challenges and vagaries of life in the context of resettlement.

As the group of participants was small, the study identified experiences that were specific to each participant, highlighted the sociocultural context defining these experiences, and elicited meaning about how the participants made sense of their nuanced lived experiences and respect for what matters to them. The identified themes all spoke to the commonalities within these lived experiences, including mental health aspects centred around protective factors of an altruistic collectivist understanding centred around their families.

Qummouh and Linnell (2023) point out the connection between the trauma and psychosocial challenges that refugees face in their homeland, the multiple internal displacements, and then moving into transit is crucial in understanding the factors that determine resilience and mental health outcomes in resettlement. These connections can help identify critical factors in how refugees’ narratives contribute to understanding the effects of trauma and displacement on their settlement in host countries as crucial considerations in therapeutic practices and policies [26].

Congruent with this psychosocial emphasis in the research, the findings of this study tentatively suggested that the participants’ experience of survival depended in significant part on constructs surrounding culture and privilege. Even among this small group of participants, it was evident that considerations of class, education, and gender had influenced participants’ modes of survival in a transit country, had set the stage for more or less successful enculturation on resettling in Australia, and continued to have consequences for their overall mental health and well-being. This reinforced insights from the literature that complex intersectionality mediates the impacts of trauma and displacement [31,32,33]. A tension emerged in the narratives between cultural and social constructs of survival and individual strengths and determination, and these factors were significantly tied with economic privilege, employment, and gendered role expectations. This finding is further supported by Kiremit and Akfirat’s (2023) study on the experience of Syrian refugee women in Turkey, revealing that the impact on refugee women’s mental health and well-being depended on perceived attitudes towards employment. Their findings go on to state that factors such as working conditions and gender and ethnic discrimination play a significant role in mental health and wellbeing outcomes [34].

Interestingly, the six female participants of this study who held professional roles such as accounting or teaching and were able to maintain their employment in Syria (as long as they possibly could) were the same participants who went on to find jobs in transit to survive and were more successful in finding employment in Australia. They described their mental well-being as significantly better than that of those participants who struggled with securing employment at home and in transit, who, in turn, described experiencing more mental health problems during their resettlement. Those participants who were from lower socio-economic and or educational levels in Syria went on to struggle to find longer-term employment in transit. They described being subjected to insecurity, sexual harassment, and exploitation in jobs such as cleaning that were poorly paid and considered to be low status. Higher levels of mental health symptoms of depression and anxiety in transit, which carried over into resettlement in Australia. These participants told the first author about clinically significant levels of depression, anxiety, and PTSD symptoms for which they are seeking psychological services. Farahani et al.’s (2021) systematic review, supports this finding, stating, “low socioeconomic and educational levels were strongly associated with mental health outcomes, as poorly educated refugees with lower incomes were at greater risks of developing adverse mental health outcomes” [35] (p. 16).

However, the link between educational levels and employment on mental health outcomes remains contested. Research conducted by Testa (2023); Dietrich et al. (2019), Campbell et al. (2018), and Segal et al. (2018) outline that factors such as language barriers and quality of accommodation, among other factors, which all seven of the participants of this study incurred, were also compounding factors contributing to mental health outcomes in resettlement [36,37,38,39]. The challenges of adjusting to new cultures and environments contributed to the loss of self-esteem for all participants and, for some, of their dignity, leaving them feeling helpless and hopeless.

This brings the study to an interesting finding surrounding cultural gender differences, given that six out of the seven participants were female. The study found similar gendered expectations in the narratives of the six women, all of whose narratives were deeply implicated in a sense of responsibility for the emotional and relational well-being of their families and especially their children. However, male privilege in Alfred’s narrative is complicated by the existential despair that can equally arise in the face of gendered and culturally normative expectations of being responsible for the family’s security when circumstances are so far outside of one’s control. These findings are not surprising or uncommon narratives of refugees. What is of more significance is that these same obligations and commitments are what sustain participants, so much so that they define ‘thriving’ as the opportunity to contribute to a better future [40,41].

This article has unpacked connections within and between the participants’ narratives of lived experiences in each stage of their journey, each with its own unique influences of experiences and challenges that had to be overcome based on their roles and responsibilities in a family and culturally collectivist context. Despite the constraining and enabling effects of these influences and experiences, the strength and depth of connection to culture, family, and spirituality are arguably the primary factors connecting the thread of hope in all seven stories.

The participants’ narratives often showed them overcoming discrimination, rising above the obstacles faced in the trajectory of their journey, and the traumas experienced throughout their displacements. Despite the profound sense of loss and sometimes loneliness that haunts these stories, the participants did not do this alone. Throughout the challenges faced by the participants in the different stages of displacement, having opportunities to secure a future that at one stage was deemed impossible, a collective cultural identity persisted. This key finding of a collective sense of culture versus the individualist sense of self and identity is what underpins the research findings beyond conventional understandings of trauma and resilience.

Amidst ongoing and dynamic conflict in the Middle East, Syria may not always be in the spotlight, yet the Syrian humanitarian crisis continues to underscore the devastating and enduring effects of war on individuals, families, and whole communities. Alongside the violent atrocities of war, significant impacts on the economy, the pressure on health services (the unimaginable attacks on roads, hospitals, and schools), and crucially, the destruction of housing infrastructure that connects personhood and identity contribute to overwhelming grief, loss, and heartbreak. Within this current context, the stories of the Syrian refugee participants are a potent reminder of the cruelty and enduring aftermath of war and of the irrepressible humanity of those who have suffered its inhumanity. There is still much to be learned from one of the most significant humanitarian crises that the world has ever witnessed. The refugee participants of this study, and those who have similarly witnessed firsthand the contexts and reality of despair, are voices we need to learn from.

## 5. Conclusions

This study contributes to the growing body of empirical research that deepens the understanding of the constructs and experiences related to displacement. The capacity to deeply understand what sustains hope for the Syrian refugees in this study is a critical aspect of research that cannot be overlooked. We now have a more nuanced understanding of the central roles that family, community, spirituality, and the experiences of the second generation, play in the well-being of resettled people. We have learned that traumatic and sustaining memories are deeply interconnected, and the way researchers listen to these stories significantly influences our understanding, leading to practical implications that support the idea that there is no one-size-fits-all approach. One thing that clinical authors and practitioners could take from this study is the importance of noticing when we slip back into assumptions that detract from the dignity and specificity of these stories and the language and metaphors in which they are told. Displacement tries to rob people of the agency to tell their own stories in their own words, and the opportunity to do this can be an emotional and embodied place of refuge in and of itself.

Through the voices of the refugees who participated in this research, resilience emerges as an individual and collective capacity in the face of ongoing challenges of displacement and resettlement. The participants’ stories show how their resilience is supported and complicated by memories of places and people left behind, shaped by care and hope for their resettled family’s current and future lives, and sustained by cultural, community, and spiritual connections.

Al-Muhannadi and Buheji (2024) have recently proposed that an understanding of refugee resilience as based on “cultural, spiritual and communal strength… serves as an inspiring model for sustaining dignity and hope in the face of overwhelming challenges” [42] (p. 27). The stories of the seven Syrian refugees contribute to this expanded understanding of resilience as they rise each day from dreaming on the pillow of tears.

## Figures and Tables

**Table 1:** Snapshot of key information regarding the home to host via temporary displacement in transit counties.

Participant	Home	Sociocultural Background/Education	Left Syria	Internally Displaced	Transit	Length of Stay in Transit	Arrival in Australia
Alina	Al Hasakah	Dental Assistant/Stay-at-home Parent High School Graduate	2011	No	Lebanon	7+ years	2018/2019
Alfred	Al Mishtai (Homs)	High School Principal/Accountant College Graduate	2017	Twice	Lebanon	1 year	2018
Maryam	Jaramana (Damascus)	Executive Legal Secretary College Graduate	2017	Multiple	Erbil	2 years	December 2019
Mona	Al Hasakah	Carer Primary School	2017	3 times	Lebanon	1 year	December 2017
Yusra	Jaramana (Damascus)	Single University Student	2017	Multiple	Erbil	2 years	December 2019
Zohra	Jaramana (Damascus)	Teacher College Graduate	2014	Multiple	Lebanon	2 years	2016
Maya	Al Mushrifah (Homs)	Accountant College Graduate	2018	Twice	Erbil	1 year	2019

## Data Availability

The data sets are not publicly available as they contain information that could potentially re-identify individuals. However, they are available from R.Q. upon reasonable request and with relevant ethical approval in place.
